# Dystrophic calcinosis cutis in a patient with cutaneous sarcoidosis in remission

**DOI:** 10.1002/ski2.174

**Published:** 2022-09-29

**Authors:** Miyuki Yoshikawa, Eijiro Akasaka, Hajime Nakano, Daisuke Sawamura

**Affiliations:** ^1^ Department of Dermatology Hirosaki University Graduate School of Medicine Hirosaki Japan; ^2^ Division of Dermatology Aomori Prefectural Hospital Aomori Japan; ^3^ Division of Dermatology Kuroishi General Hospital Kuroishi Japan

## Abstract

A 65‐year‐old Japanese woman was referred to our department because of a 5‐month history of asymptomatic papules on the face. She was diagnosed with cutaneous sarcoidosis on the face 20 years ago. All of the lesions had completely disappeared with oral corticosteroids. Twenty years after the diagnosis of sarcoidosis, small papules developed in areas where the cutaneous sarcoidosis had been located. Physical examination revealed four yellow‐white papules on the face. Dermoscopy revealed a homogenous, round, and yellow‐white lesion. Serum levels of calcium and phosphorus were normal. Histopathology demonstrated calcium deposits in the dermis surrounded by inflammatory infiltrates without sarcoid granulomas. We made a diagnosis of calcinosis cutis. Basal cell carcinoma with calcinosis cutis, milia‐like calcinosis cutis, and subcutaneous calcified nodule should be differentiated. Calcinosis cutis can be classified into four subtypes based on pathogenesis: dystrophic, metastatic, idiopathic, and iatrogenic. Dystrophic calcinosis cutis is caused by local tissue damage or abnormalities. Whereas, metastatic calcinosis cutis is often associated with hypercalcaemia, hyperphosphatemia, or hyperparathyroidism. There are reported cases of metastatic calcinosis cutis associated with sarcoidosis because patients with sarcoidosis often present with hypercalcaemia. However, dystrophic calcinosis cutis associated with sarcoidosis has been rarely reported. In the present case, systemic treatment for sarcoidosis may have degraded sarcoid granulomas and yielded necrotic tissue and dermal fibrosis, which might have induced ectopic calcification. Thus, we thought the present case consisted of dystrophic calcinosis cutis that developed in areas with cutaneous sarcoidosis in remission.

## Graphical abstract

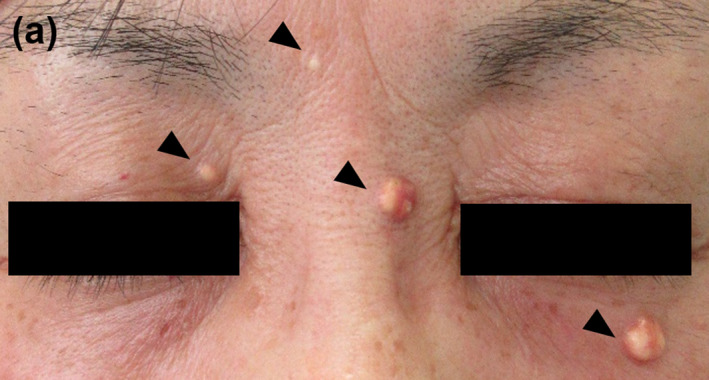

We present a case of dystrophic calcinosis cutis on the face. They developed in areas with cutaneous sarcoidosis in remission.

## 1 CASE REPORT

**FIGURE 1:**
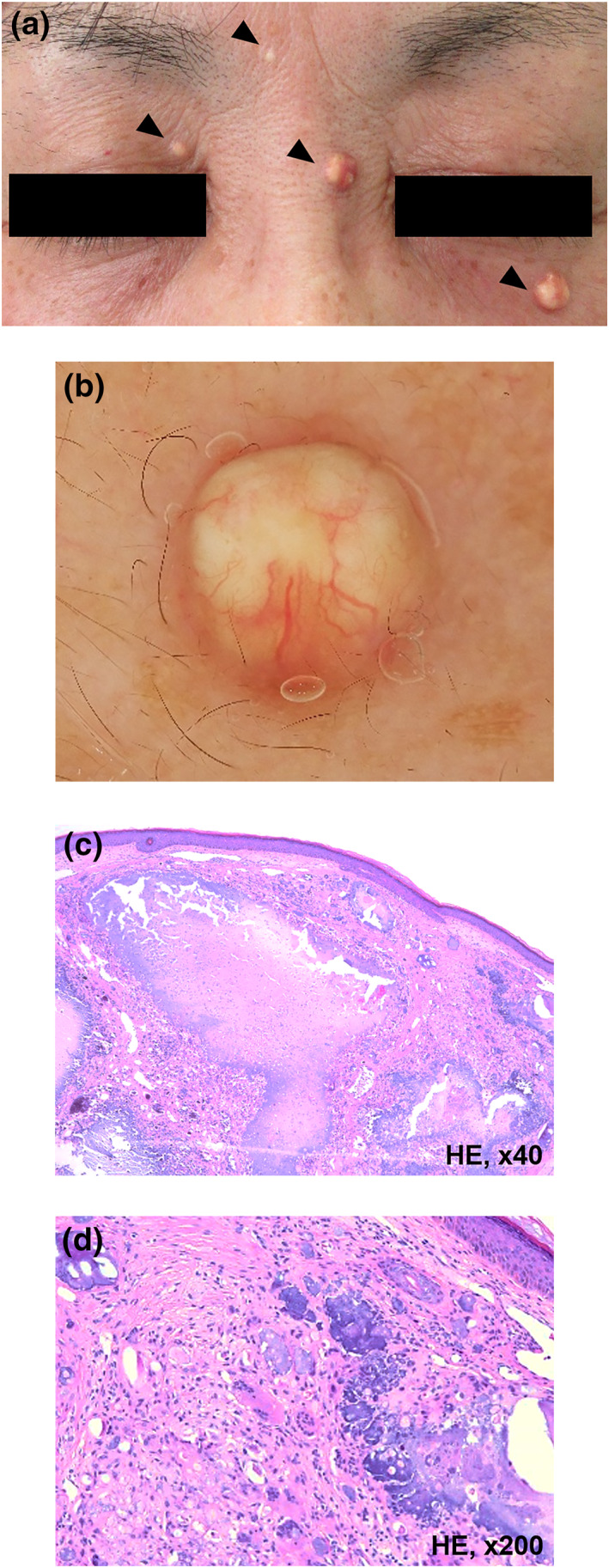
(a) Clinical manifestations of calcinosis cutis. There were four dome‐shaped yellow‐white papules measuring 1–5 mm in diameter around the eyes and eyebrows (arrowheads). (b) Dermoscopy showed a homogenous, round, and yellow‐white lesion and telangiectasis. (c) Histopathologic examination showed calcification with basophilic material in the dermis (Haematoxylin‐eosin (HE) staining, original magnification ×40). (d) In the high‐power view, there were many multinucleated giant cells and dermal fibrosis (HE staining, ×200)

A 65‐year‐old Japanese woman presented to our outpatient clinic with a 5‐month history of asymptomatic papules on the face. She was diagnosed with sarcoidosis 20 years ago based on cutaneous sarcoid lesions, uveitis, and bilateral hilar lymphadenopathy. The skin lesions were 5–10 mm red‐brown or violaceous papules distributed around the eyelid and nose. Oral prednisolone successfully decreased those symptoms; all of the cutaneous sarcoid lesions completely disappeared. Twenty years after the diagnosis of sarcoidosis, she noticed small papules that developed in areas where the cutaneous sarcoid lesions had been located. Each lesion had gradually enlarged. She had been taking low‐dose oral prednisolone (5 mg/day) at the presentation. Physical examination revealed four yellow‐white papules on the face. They were well‐demarcated, firm, and 1–5 mm in diameter (Figure 1a). Dermoscopy revealed a homogenous, round, and yellow‐white lesion on the left lower eyelid with telangiectasia on the surface (Figure 1b). Laboratory testing revealed normal serum levels of calcium, phosphorus, and parathyroid hormone. Histopathology of the papules demonstrated deposits of basophilic material in the dermis surrounded by lymphohistiocytic inflammatory infiltrates and multinucleated giant cells without sarcoid granulomas (Figure 1c,d). In addition, no characteristic findings of BCC such as aggregates of basaloid cells with peripheral palisading or retraction artefact were observed in the sections. Based on these findings, we made a diagnosis of calcinosis cutis in the areas with cutaneous sarcoidosis in remission.

Given the patient's age, location of the skin lesions, and dermoscopic findings, BCC would be an important differential diagnosis in this case. It has been reported that the calculated incidence of BCC with calcinosis cutis is 14%.^1^ In addition, benign tumours associated with hair follicles such as pilomatricomas, trichilemmal cysts, and trichoepitheliomas also display calcinosis cutis.^2^ Even when initial superficial histological sections of a tissue specimen revealed only calcium deposition in the dermis, malignant or benign tumours with calcinosis cutis could be found in deeper section.^1^ Therefore, we made deeper sections and carefully reconfirmed that there was no histological finding of BCC or other tumours. In addition, idiopathic milia‐like idiopathic calcinosis cutis (MICC) and subepidermal calcified nodule (SCN) are also important differential diagnoses. Milia‐like idiopathic calcinosis cutis is a rare type of idiopathic calcinosis cutis that mostly occur in childhood and is associated with Down syndrome in about two thirds of the patients. It is characterized by 1–4 mm in diameter, whitish papules similar to milia. The upper and lower extremities are most commonly affected, whereas involvement of the face has rarely been reported.^3^, ^4^ SCN manifests as an asymptomatic, white to yellowish papulae or nodule with a smooth or verrucous surface. It develops as a solitary lesion on the face, but multiple lesions and involvement of extremities have been occasionally reported. Histologically, it usually shows an epidermal reaction such as hyperkeratosis, focal parakeratosis, and acanthosis in addition to dermal calcium deposits.^4^, ^5^, ^6^ It is very difficult to distinguish these diseases from dystrophic calcinosis cutis owing to similar clinical manifestations. However, in the present case, calcinosis occurred in the area where cutaneous sarcoidosis had been located. In addition, histological examination revealed no associated epidermal changes that are seen in SCN. Taken together, we made a provisional diagnosis of calcinosis cutis rather than MICC and SCN.

Calcinosis cutis can be classified into four subtypes based on pathogenesis: dystrophic, metastatic, idiopathic, and iatrogenic. Dystrophic calcinosis cutis, the most common subtype, is caused by local tissue damage or abnormalities due to trauma, infection, tumour, connective tissue disorder, or other conditions. Serum calcium and phosphate levels are normal. In contrast, metastatic calcinosis cutis is often associated with hypercalcaemia, hyperphosphatemia, or hyperparathyroidism. Idiopathic calcinosis cutis occurs in the absence of tissue damage or metabolic abnormalities. Iatrogenic calcinosis cutis is caused by a medical treatment or procedure.^7^ Sarcoidosis is a systemic granulomatous disorder of unknown aetiology. Cutaneous manifestations of sarcoidosis can be divided into two categories: specific, and non‐specific cutaneous sarcoidosis. The former features sarcoid granulomas in the dermis, while the latter is characterized by secondary reactive inflammatory processes such as calcinosis cutis and erythema nodosum.^8^ Some reported cases of metastatic calcinosis cutis were associated with sarcoidosis; that may be because 20% of patients with sarcoidosis present with hypercalcaemia. In contrast, dystrophic calcinosis cutis has been rarely reported in patients with sarcoidosis. Some dystrophic calcinosis cutis lesions develop in cutaneous sarcoidosis lesions while others arise in non‐affected areas.^9^, ^10^

The pathophysiology of dystrophic calcinosis cutis remains controversial. It has been suggested that phosphate‐binding protein released from necrotic cells induces phosphate deposition and consequent calcification at sites of trauma and inflammation.^7^ In addition, Reiter et al. hypothesized that abnormal changes in collagen, elastic fibres, and subcutaneous fat might lead to ectopic calcium deposition.^7^, ^11^ This patient had cutaneous sarcoidosis on the face 20 years ago and histologic examination showed fibrosis around the dystrophic calcinosis cutis lesion. We believe that systemic treatment for sarcoidosis degraded sarcoid granulomas and yielded necrotic tissue, which was followed by inflammation and dermal fibrosis in the area. Necrosis and fibrosis might have induced ectopic calcification. Thus, we thought the present case consisted of dystrophic calcinosis cutis that developed in areas with cutaneous sarcoidosis in remission.

## CONFLICT OF INTEREST

The authors declare that there is no conflict of interest that could be perceived as prejudicing the impartiality of the research reported.

## AUTHOR CONTRIBUTIONS

**Miyuki Yoshikawa**: Conceptualization (lead); Data curation (lead); Formal analysis (lead); Investigation (lead); Methodology (lead); Visualization (supporting); Writing – original draft (lead); Writing – review & editing (supporting). **Eijiro Akasaka**: Conceptualization (lead); Investigation (equal); Supervision (lead); Validation (lead); Visualization (lead); Writing – original draft (supporting); Writing – review & editing (lead). **Hajime Nakano**: Conceptualization (equal); Investigation (supporting); Supervision (supporting); Validation (supporting); Writing – review & editing (equal). **Daisuke Sawamura**: Conceptualization (supporting); Investigation (supporting); Supervision (supporting); Validation (supporting); Writing – review & editing (equal).

## ETHICS STATEMENT

The patient provided informed consent for publication, including for use in social media.

## Data Availability

Data sharing is not applicable to this article as no new data were created or analysed in this study.
